# Normal trajectory of Interleukin-6 and C-reactive protein in the perioperative period of total knee arthroplasty under an enhanced recovery after surgery scenario

**DOI:** 10.1186/s12891-020-03283-5

**Published:** 2020-04-21

**Authors:** Ze Yu Huang, Qiang Huang, Li Ying Wang, Yi Ting Lei, Hong Xu, Bin Shen, Fu Xing Pei

**Affiliations:** 1 Department of Orthopedic Surgery, West China Hospital, West China Medical School, SiChuan University, 37# Wainan GuoXue Road, ChengDu, SiChuan Province People’s Republic of China; 2 Department of Operation Room, West China Hospital, West China Medical School, SiChuan University, ChengDu, SiChuan Province People’s Republic of China

**Keywords:** Total knee arthroplasty, Enhanced recovery after surgery, ERAS, CRP, IL-6

## Abstract

**Background:**

We designed the current study to understand the normal trajectories of interleukin-6 (IL-6) and C-reactive protein (CRP) in the immediate hours and days after primary total knee arthroplasty (TKA) under the management of an enhanced recovery after surgery (ERAS) protocol and examined whether one or the other returned to normal more quickly.

**Methods:**

In this prospective cross-sectional study, we examined the plasma IL-6 and CRP levels in 100 patients undergoing primary TKA at the following time points: 12 h preoperatively as well as postoperatively 12 h, 48 h, 3 days and 2 weeks. Patients were followed up for 1 year to monitor the postoperative complications, especially the infection.

**Results:**

IL-6 peaked at 48 h postoperatively. Then IL-6 started to decline at 3 days postoperatively and went back to baseline level at 2 weeks (*p* = 0.950). CRP peaked at 3 days postoperatively. At 2 weeks, CRP declined to a normal range, without being significantly different from the baseline level (*p* = 0.816).

**Conclusion:**

We found that under the ERAS scenario, the postoperative peak of IL-6 and CRP was deferred compared with previous studies. Compared to IL-6, CRP showed a gradual rise after surgery. Both of these two biomarkers returned to normal under the ERAS scenario. Future multiple-center studies with larger sample size can help define the thresholds of IL-6 and CRP for periprosthetic joint infection (PJI) early diagnosis. With these reference data, a clinician can make a quicker decision to perform aspiration to diagnose early PJI and benefits more patients.

## Background

Periprosthetic joint infection (PJI) is a major complication after total knee arthroplasty (TKA). The decisive importance for the successful therapy of a PJI lies in an early and reliable diagnosis, which enables quick initiation of therapy and may make it possible to salvage the prothesis. However, the early clinical manifestation of PJI may be nonspecific and is becoming a challenge plague for the joint surgeons all over the world. Serological tests, including erythrocyte sedimentation rate (ESR), C-reactive protein (CRP) and Interleukin-6 (IL-6), are the simplest and most routine investigation into a guide for early PJI [1].

Though CRP and IL-6 have been proved to be of high sensitivity but relatively low specificity [1, 2]. Both of the two biomarkers have been established as parameters assessing inflammation after TKA [3–7]. As for CRP, it is hard to interpret early PJI because studies have shown that CRP will elevate as long as 3 months after the surgery even in the absence of infection [7]. IL-6, a major endogenous protein mediator of the acute phase inflammation, returns to normal level soon after surgery [1, 2, 8]. Studies have shown that IL-6 not only elevated because of surgical inflammation [4] but also in late PJI. Recently, Maniar et al. [9] have investigated the serum levels of IL-6 and CRP in the hours and days immediately postoperatively to understand their normal trajectory. However, with the promotion of enhanced recovery after surgery (ERAS), many methods have been used to minimize surgical trauma and reduce inflammation [3, 10]. Little is known of the normal curve in the hours and days of CRP and IL-6 immediately postoperatively under an ERAS scenario.

We therefore undertook this prospective study in 100 patients undergoing primary TKA for osteoarthritis to define the normal trajectory of IL-6 and CRP over a 2-week period of patients receiving ERAS process. We presented our results at five time points, namely 12 h preoperatively and postoperatively at 12 h, 48 h, 72 h, and 2 weeks respectively. We have attempted to find out whether IL-6 and CRP levels returned to baseline during this study period.

## Methods

The current prospective cross-sectional study was approved by our institution’s ethical committee (No. 201302009) and conducted between February and May 2018. Written informed consent was obtained from the patients before their participation in the study.

Between February and May 2018, 172 consecutive patients underwent primary TKA performed by the surgical team. Those who had comorbidities known to affect serum IL-6 and CRP such as inflammatory arthritis, autoimmune disorders, history of cancer or long-term steroid intake were excluded. Individuals were excluded because of inflammatory arthritis (38 patients), lung cancer (two patients) and unwillingness to participate (32 patients).

Patients age ranged from 48 to 85 years (67.8 ± 7.9 years); body mass index (BMI) ranged from 17.9 to 33.78 kg/m^2^ (25.5 ± 3.4 kg/m^2^). Of the included patients, there were 13 male (13%) and 87 female (87%). All TKAs were performed with the patients under general anesthesia by one surgical team composed of two senior orthopedic surgeons. The operations were done in the standard way, using a midline skin incision, a standard medial parapatellar approach and a measured resection technique. The standard intraoperative ERAS protocol for TKA was neither using tourniquet nor drainage [3]. Intravenous tranexamic acid was used 5 to 10 min before the skin incision (20 mg/kg) and 3, 6, 12, and 24 h later (10 mg/kg) along with 1 g of topical tranexamic acid (TXA) in 50 mL of normal saline solution. Periarticular infiltration, 150 mg ropivacaine and 0.2 μg epinephrine diluted in 60 mL of normal saline, was used for pain management along with postoperative oral diclofenac sodium (Voltaren; 50 mg twice daily). All patients were evaluated by a physical therapist and began walking with partial weight-bearing and a knee brace to protect the surgical site on the day of the surgery and 3 times daily thereafter until hospital discharge. A combination of mechanical and chemical prophylaxis was adopted to prevent venous thromboembolism. An intermittent foot-pump system was used as a routine practice to prevent deep venous thrombosis (DVT) before the patient began walking. Half-dose enoxaparin (Clexane; 0.2 mL containing 2000 IU) was administered subcutaneously 6 h postoperatively, and a full dose (0.4 mL containing 4000 IU) was given at 24-h intervals until discharge. After discharge, 10 mg of rivaroxaban was administered orally for 10 days if no bleeding events occurred. All patients were discharged 3 days postoperatively. Doppler ultrasound was used to evaluate for DVT at the time of discharge and at 1, 3-month follow-up evaluation.

A complete blood-cell count along with CRP and IL-6 was measured 12 (±1) hours preoperatively and postoperatively at 12 (±1) hours, 48 (±2) hours, 72 (±3) hours, and 2 weeks (±6 h) respectively. All the measurements of biomarkers were done by the department of laboratory medicine of our hospital certificated by CAP (Clinic American Pathology). Assessment of serum IL-6 was done using the electrochemiluminescence immunoassay (Cobas E601 analyzer; Roche Diagnostics GmbH, Mannheim, Germany) (normal range, 0–7 pg/mL). Quantitative assessment of CRP was done using an immunoturbidometric assay (Cobas analyzer; Roche Diagnostics GmbH) (normal value < 5.0 mg/L). For the purpose of statistical analyses, any value below the lowest limit of detection (LLOD) for the biomarker assay was imputed as 1/2 LLOD of the assay. Patients were followed up at 2 weeks, 3 months, 6 months and 1 year respectively. In our previous study [11], we found that ESR is a less sensitive parameter compared to CRP and IL-6. In addition, the value of the ESR changed according to the two different diagnostic kits used in our institute. Thus, we did not include ESR as a parameter in the current study.

Data were managed and analyzed by SPSS 25.0 for Mac (SPSS Inc., Chicago, IL, USA). The values of IL-6 and CRP were displayed as scatter plots with 95% confidential interval as error bars using software GraphPad Prism 8.0 (GraphPad Software, Inc., San Diego, CA). Owing to the failure of the normality test (Shapiro-Wilk test); Friedmann’s one-way repeat-measures analysis of variance was applied, giving *p* < 0.001 for both IL-6 and CRP. Post hoc Dunnett’s test, an extension of the Friedman repeated-measures test, was applied to compare each pairwise data to find out which pair differed from baseline.

## Results

### Il-6

**Fig. 1:**
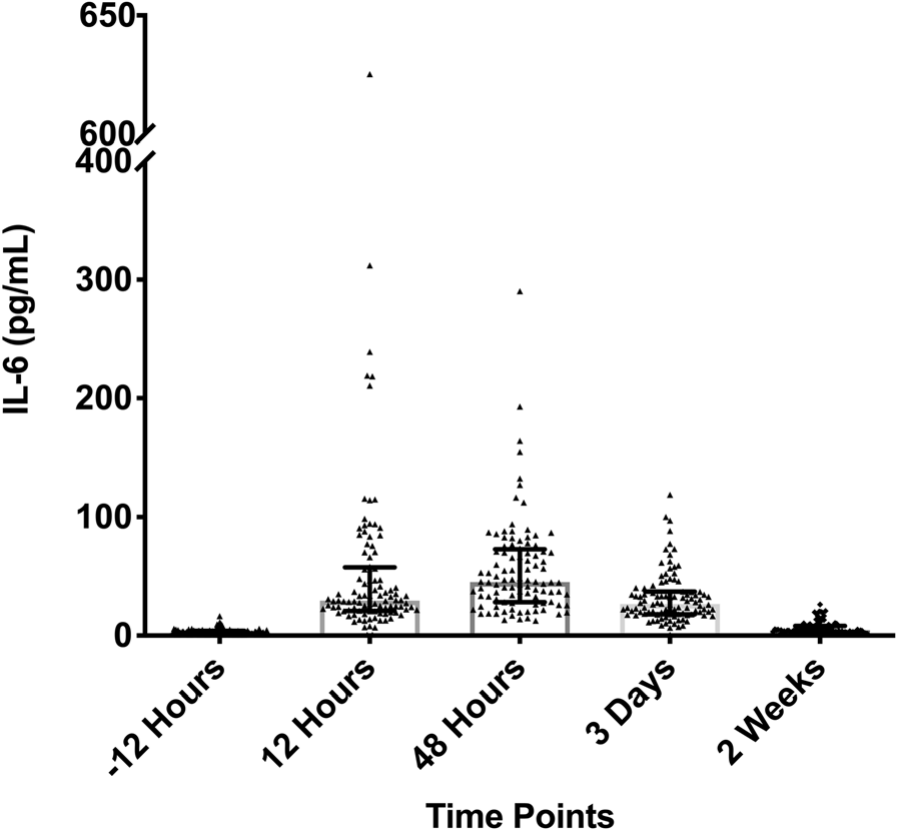
Trend of IL-6 from preoperative to 2 weeks postoperatively is shown. The error bars indicate the mean with 95% confidence interval

The results of IL-6 showed a sharp rise in its value from baseline to peak as early as 48 h postoperatively and then started to decline at 3 days. By 2 weeks after the surgery, IL-6 values had reached baseline levels (Fig. 1). The preoperative baseline mean value was 3.43 ± 2.44 pg/mL (range, 1.50–16.34 pg/mL) (Table 1). At 12 h postoperatively, the levels of IL-6 reached mean value of 54.48 ± 77.87 pg/mL (range, 1.5–625.20 pg/mL). At 48 h, it rose to the peak mean value of 56.29 ± 41.20 pg/mL (range, 12.43–290.40 pg/mL). At 72 h, it started to decrease to a mean value of 32.23 ± 21.33 pg/mL (range, 0.00–118.9 pg/mL). At 2 weeks, the mean value of IL-6 went back to 6.62 ± 5.21 pg/mL (range, 1.50–26.2 pg/mL), without being different from the preoperative mean value (*p* = 0.950) (Table 2).

**Table 1:** IL-6 and CRP levels over the study period

Parameter	Time	Mean (SD)	95% CI	IQR	Median	Range
IL-6 (pg/mL)	12 h preoperative	3.28 ± 2.57	2.77–3.80	2.26	2.80	0.75–16.34
	12 h postoperative	54.46 ± 77.88	39.01–69.92	36.90	29.46	0.75–625.20
	48 h postoperative	56.29 ± 41.20	48.12–64.47	44.75	45.17	12.43–290.40
	3 days postoperative	32.23 ± 21.32	28.00–36.46	18.96	26.62	0.75–118.90
	2 weeks postoperative	6.59 ± 5.24	5.55–7.63	4.89	4.55	0.75–26.20
CRP (mg/L)	12 h preoperative	3.35 ± 2.11	2.93–3.77	1.85	2.67	1.02–16.20
	12 h postoperative	25.17 ± 20.47	21.11–29.24	20.08	17.80	1.35–84.90
	48 h postoperative	66.57 ± 43.11	58.02–75.12	63.02	58.85	6.39–198
	3 days postoperative	75.98 ± 40.01	68.04–83.92	50.45	76.65	0.5–196
	2 weeks postoperative	6.67 ± 5.23	5.64–7.71	4.64	4.87	1.54–30.90

*IL-6* interleukin-6, *CRP* C-reactive protein, *SD* standard deviation, *CI* confidence interval, *IQR* interquartile range

**Table 2:** Trend of IL-6 and CRP: comparisons of baseline and postoperative levels

Study parameter	Group	Mean Difference	Multiple comparisons (Dunnett’s method) with preoperative
IL-6 (pg/mL)	Preoperative	–	–
	12 h	51.18	< 0.001
	48 h	53.01	< 0.001
	3 days	28.95	< 0.001
	2 weeks	3.30	0.943
CRP (mg/L)	Preoperative	–	–
	12 h	21.82	< 0.001
	48 h	63.22	< 0.001
	3 days	72.63	< 0.001
	2 weeks	3.32	0.816

Normality test (Shapiro-Wilk test) failed; thus, Friedman’s one-way repeat-measures analysis of variance was applied, which gave *p* < 0.001 for both IL-6 and CRP; post hoc Dunnett’s test, which is an extension of the Friedman RM test, compared each pairwise data to find which pair differed from baseline; *IL-6* interleukin-6, *CRP* C-reactive protein, *RM* repeat measure

### CRP

**Fig. 2:**
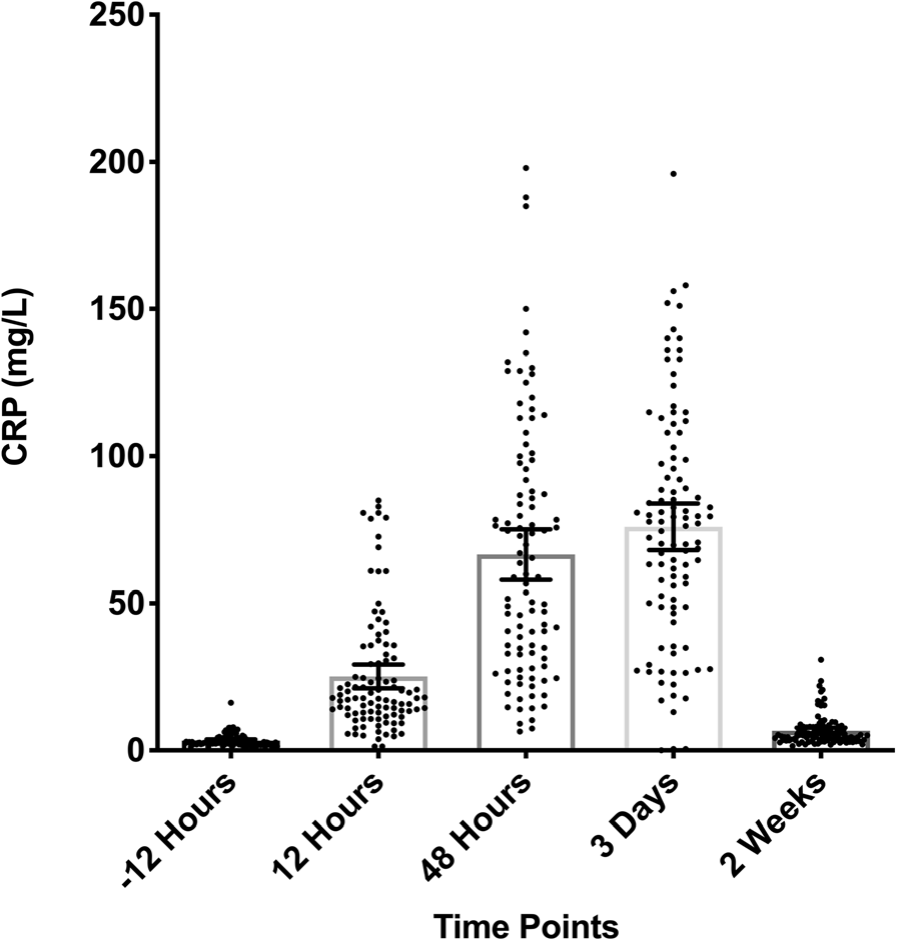
Trend of CRP from preoperative to 2 weeks postoperatively is shown. The error bars indicate the mean with 95% confidence interval

The CRP results showed its value peak at 72 h postoperatively and then declined. At 2 weeks, CRP levels almost returned to baseline (Fig. 2). The preoperative baseline mean CRP value was 3.35 ± 2.11 pg/mL (range, 1.02–16.20 pg/mL) (Table 1). At 12 h postoperatively, the CRP values increased to a mean value of 25.17 ± 20.47 pg/mL (range, 1.35–84.90 pg/mL). By 48 h after TKA, the CRP level had risen furtherly to a mean value of 66.57 ± 43.11 pg/mL (range, 6.39–198.00 pg/mL). At 72 h postoperatively, the levels of CRP reached its peak mean value of 75.97 ± 40.04 pg/mL (range, 0.00–196 pg/mL). At 2 weeks, CRP levels almost returned to baseline with a mean value of 6.67 ± 5.23 pg/mL (range, 1.54–30.90 pg/mL), without being significantly different from the preoperative mean value (*p* = 0.816) (Table 2).

### Complications

Within one-year follow-up, none of the patients have any signs of infection. Neither DVT nor PE occurred in any patient. Postoperatively routine Doppler ultrasound showed that 21 patients developed intramuscular venous thrombosis. Wound secretion occurred in 3 patients during the entire follow-up period. Blistering was reported in 2 patients.

## Discussion

Though TKA has been proved a successful procedure with large improvement in patient’s quality of life [12], PJI is still a major complication after TKA with a high impact on their well-being [13]. Surgical debridement as well as antibiotics and implant retention is recommended for early PJI, being less involved and simpler than the two-stage revision for late PJI [14]. Thus, it is crucial to diagnose PJI within 3 weeks of its occurrence [15]. Once suspected by its’ clinical symptoms, the first line is to apply serologic tests. General inflammatory markers, including serum ESR and CRP are well-established markers of PJI, but it is hard to interpret their increase shortly within several weeks postoperatively because of the surgical trauma [2]. Bauer et al. [2] showed that CRP increase arising from the surgery without infection could last as long as 3 months. According to Kishimoto et al.’s [16] research, IL-6 can induce CRP synthesis. Thus, IL-6 is supposed to elevate before CRP. In addition, emerging evidences have shown that IL-6 levels are elevated in late PJI [1, 4, 17, 18]. With the ERAS procedure widely conducted in TKA, inflammation caused by surgical trauma has largely decreased [3, 19]. Till now, little is known about its normal trajectory in the days immediately after surgery, especially under an ERAS scenario [20, 21]. Thus, we undertook this prospective study to understand the normal trajectory of the two mostly used biomarkers, IL-6 and CRP after surgery.

During the inflammatory response, Il-6 is produced by monocytes and macrophages [4, 17, 22]. During infection, the serum levels of IL-6 can rise to 30–340 pg/mL [21]. Lying in the upstream of other inflammatory biomarkers, in the acute phase of inflammatory cascade, IL-6 is considered as a faster and more sensitive marker for detection of PJI [23, 24]. However, to our best knowledge, there is litter knowledge on the normal trend of IL-6 in the immediate perioperative period after primary TKA. Honsawek et al. [25] have explored the change of serum IL-6 levels in their study of 49 patients undergoing primary TKA at time points of preoperative, 24-h, 2, 4, 14 and 26 weeks after surgery. They found that the IL-6 levels reached its peak at 24-h postoperatively and returned to baseline level at 2 weeks. Their study did not explore the early time points (several days postoperatively). Wirtz et al. [21] measured IL-6 levels at preoperative, immediate postoperative, 6-, 12-, 24- and 48 h as well as 3, 4, 5, 6 and 7 days after TKA in 10 patients. They found that IL-6 reached its peak at 12 h, then declined to almost baseline level on postoperative day 4. More recently, Maniar et al. [9] measured the IL-6 trend at preoperative, 12 -, 48 h, 4 days and 2 weeks after primary TKA. They also found the IL-6 reached its peak at 12 h postoperatively and returned to baseline at 2 weeks. Our results have shown that under the ERAS scenario, the postoperative IL-6 levels reached its peak at 48 h postoperatively, being quite different from the results of previous studies. There are several possible reasons underlying the deferred IL-6 peak observed in our study. First, we avoided tourniquet use during the surgery. Thus, the increase of IL-6 caused by ischemia-reperfusion injury [26], which was induced by tourniquet, did not occur in our patients. Second, all patients in our study received intravenous and topical TXA. Several studies have shown that TXA can attenuate inflammatory responses through blockade of fibrinolysis [27–29]. As to the returning point of IL-6 to baseline, we found similar results as previous studies did.

**Fig. 3:**
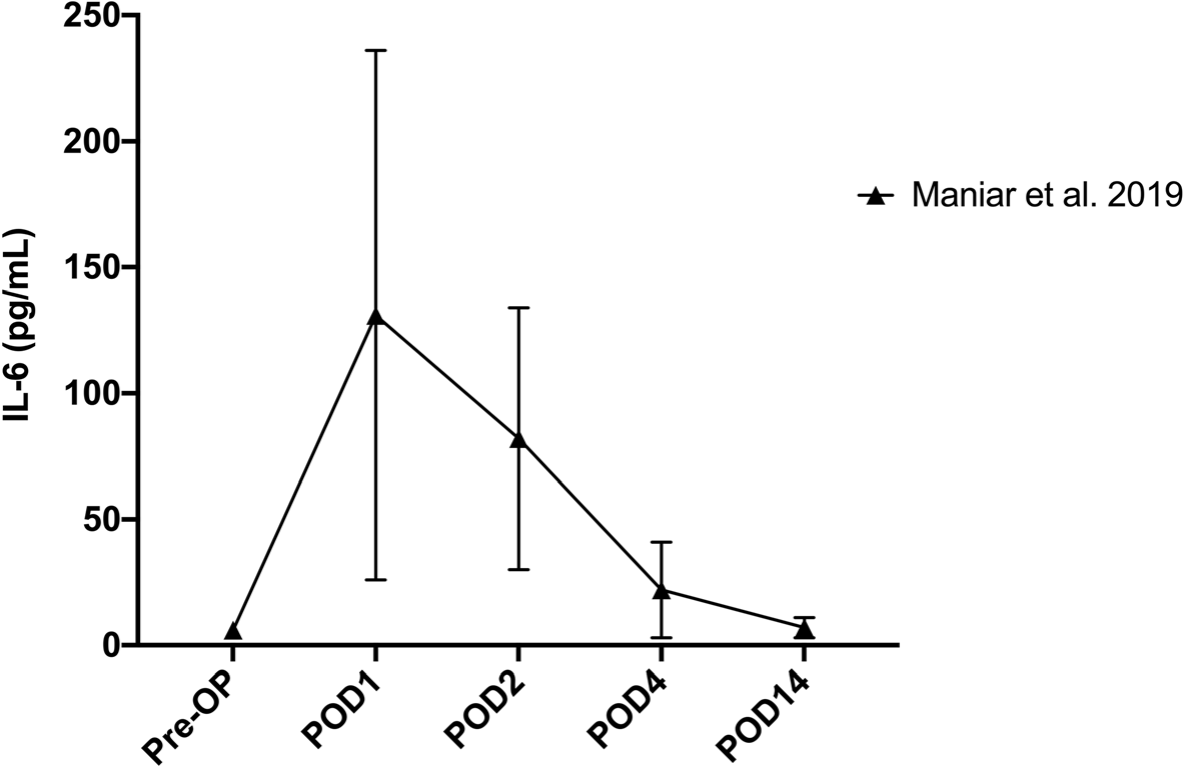
Trend of IL-6 from preoperative to 2 weeks postoperatively reported in previous study. The error bars indicate the median with interquartile range (Data were extracted from the previous study)

**Fig. 4:**
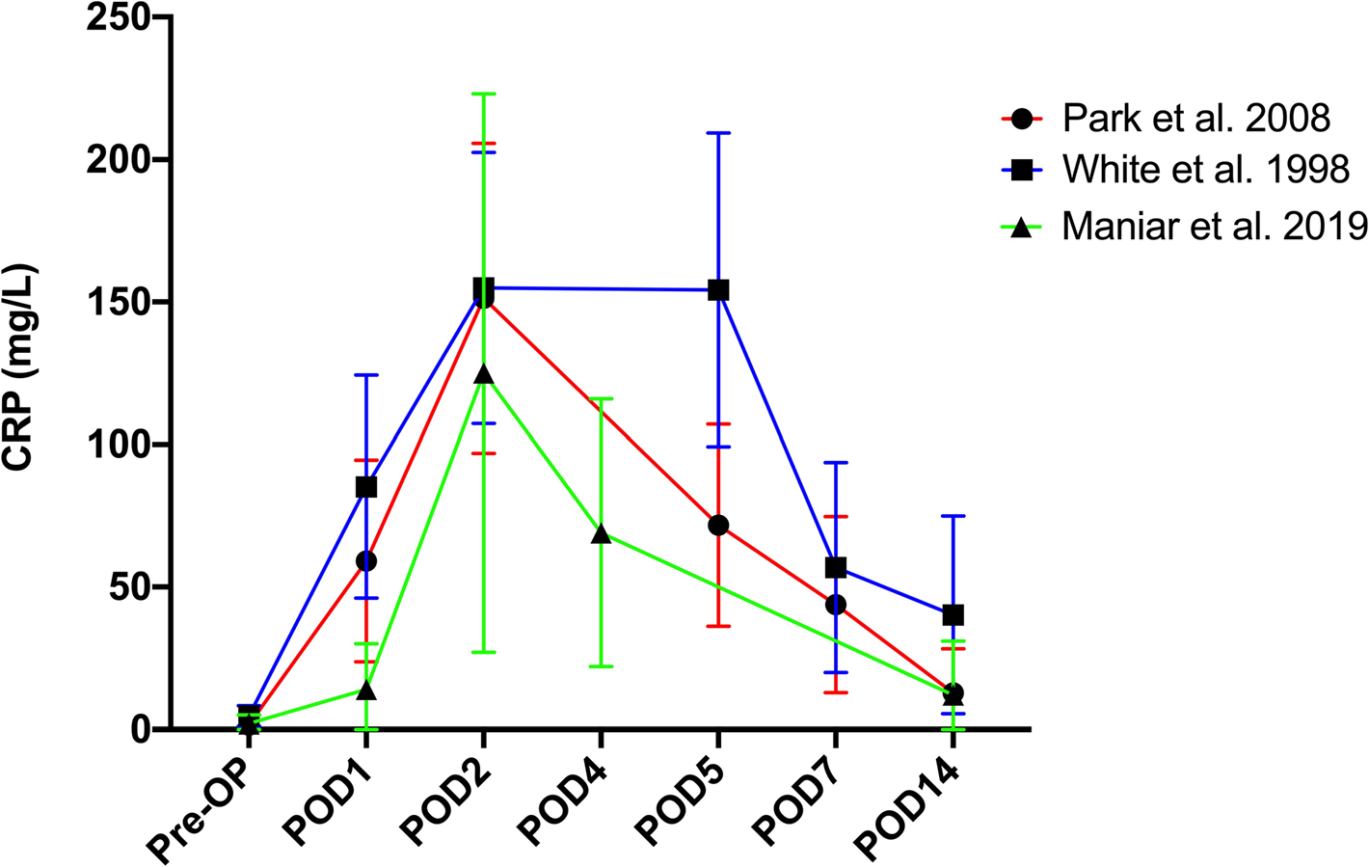
Trend of CRP from preoperative to 2 weeks postoperatively reported in previous studies. The error bars indicate the mean with standard deviation except for Maniar et al. 2019 which indicates the median with interquartile range (Data were extracted from the previous studies)

Several studies have established the trend of CRP levels after primary TKA [6, 7, 9, 30] (Table 3) (Figs. 3 & 4). White et al. [7] measured CRP in 13 patients undergoing primary TKA and found CRP levels peak at 48 h postoperatively and returned to normal at 2 weeks. However, the CRP levels at 2 weeks were still higher than the baseline. In a study including 320 patients undergoing primary TKA, Park et al. [6] found that CRP levels rose to peak values at 48 h and declined within 2 weeks after surgery. Then the CRP levels reached normal range at 6 weeks and returned to preoperative level at 3 months. Maniar et al. [9] also found that CRP levels reached its peak at 48 h postoperatively and gradually declined, and at 2 weeks, they were still higher than baseline values. In the current study, we found under ERAS scenario, the CRP levels also peaked at 48 h. While at 2 weeks postoperatively, CRP levels returned to baseline level. This is much faster than those reported in the previous studies. The main reason is probably that less surgical trauma is caused under ERAS scenario.

**Table 3:** Summary of characteristics of previous studies reporting normal trajectory of IL-6 and CRP after TKA

Study	Parameters	Number of patients	Tourniquet usage	TXA usage
White et al. 1998	CRP	108	✓	×
Park et al. 2008	CRP	13	✓	×
Maniar et al. 2019	CRP, IL-6	55	✓	×

*IL-6* interleukin-6, *CRP* C-reactive protein, *TKA* total knee arthroplasty, *TXA* tranexamic acid

All patients discharged 3 days postoperatively. The length of stay depends not only on the recovery but also on the society, culture and insurance policy. Studies have reported the prolong hospitalization differences between China and western countries. The possible reasons are as follows: First, structure of healthcare financing, including insurance type, insurance ownership, and reimbursement mechanism, may play an important role. For example, a diagnosis related group-based payment system in the United States predetermines reimbursements for hospital charge, causing early discharge. Moreover, the reimbursement ratio for inpatient service is much higher than that in the outpatient setting in China, prolonging the hospitalized length of stay [31, 32]. Second, Chinese doctors are inclined to be conservative in terms of discharge to avoid risks from vital complications, patients, or their families. Third, posthospitalization care, like nursing homes, home health, and muscle rehabilitation for patients undergoing TKA, is common in the United States whereas postoperative care needs improvements in China. Another thing needed to be emphasized here is that the core idea of ERAS lies on the early recovery of joint function other than reduced length of stay.

Limitations of the current study are as follows: First, these two biomarkers have a poor specificity [2]. Serum IL-6 and CRP are indicators of inflammatory activities including septic and non-septic [18]. However, they are still the first line of investigation when early PJI is suspected. The levels of CRP and IL-6 can be used for indications of early stage or ruling out the infection without any delay in treatment. Second, demographics of the patients including age, BMI and comorbidities were not controlled in the current study though we excluded patients with conditions, which could possibly affect serum IL-6 and CRP levels. And to our best knowledge, there were no solid evidence showing that age, BMI or comorbidities could affect these two biomarker levels in serum. Third, we excluded 24 h after the surgery, which could miss an earlier peak. Fourth, we did not use tourniquet in all of our patients. Because there is a controversy on whether tourniquet should be used in TKA, TKA without tourniquet is still not a standard care. Also, we used a specific perioperative protocol such as using TXA, specific periarticular injection and Voltaren. Thus, our results might not be applied to the most common total joint arthroplasty scenario. In addition, we only followed up for 1 year. We cannot comment on these values in recognition of late PJI. Also, PJI is a relatively rare complication after TKA with a rate of 1–2%. Therefore, the sample size of the current study is not enough to determine the threshold of PJI diagnose. Future large, multicenter studies are needed.

## Conclusion

In conclusion, our study has demonstrated the normal trajectory of IL-6 and CRP in the immediate hours and days after primary TKA under the ERAS scenario. Future large, multicenter studies are needed to establish the definitive thresholds for these biomarkers. Also, data on the confirmed cases of early PJI should be collected and analyzed to establish the predictive values of IL-6 and CRP.

## Data Availability

The datasets used and analyzed during the current study are available from the corresponding authors on reasonable request.

## Abbreviations

IL-6: Interleukin-6
CRP: C-reactive protein
TKA: Total knee arthroplasty
ERAS: Enhanced recovery after surgery
PJI: Periprosthetic joint infection
ESR: Erythrocyte sedimentation rate
TXA: Tranexamic acid
BMI: Body mass index
DVT: Deep venous thrombosis
CAP: Clinic American Pathology
